# Cyclo­art-24-ene-3*β*,26-diol from the leaves of *Aglaia exima*

**DOI:** 10.1107/S1600536810029168

**Published:** 2010-07-31

**Authors:** Khalijah Awang, Xe Min Loong, Khalit Mohamad, Soon Lim Chong, Seik Weng Ng

**Affiliations:** a Department of Chemistry, University of Malaya, 50603 Kuala Lumpur, Malaysia; b Department of Pharmacology, University of Malaya, 50603 Kuala Lumpur, Malaysia

## Abstract

Cyclo­art-24-ene-3*β*,26-diol, C_30_H_50_O_2_, isolated from the leaves of *Aglaia exima*, has three six-membered rings fused together that adopt chair conformations. There are two independent mol­ecules in the asymmetric unit. O—H⋯O hydrogen bond inter­actions between the hydroxyl groups in the 3*β* and 26 positions lead to the formation of a layer structure parallel to (10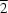).

## Related literature

For the spectroscopic characterization of the title compound, see: Anjaneyulu *et al.* (1985 ▶, 1994 ▶); Parveen *et al.* (1990 ▶); Takahashi & Takani (1975 ▶).
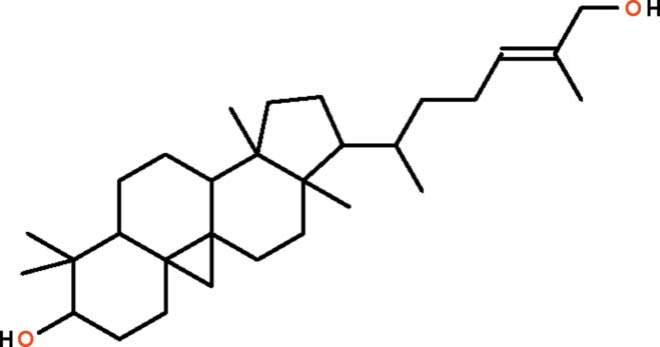


## Experimental

### 

#### Crystal data

C_30_H_50_O_2_
*M* *_r_* = 442.70
Monoclinic, 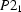

*a* = 9.9950 (14) Å
*b* = 7.512 (1) Å
*c* = 34.820 (5) Å
β = 91.029 (2)°
*V* = 2614.0 (6) Å^3^
*Z* = 4
Mo *K*α radiation
μ = 0.07 mm^−1^
*T* = 100 K
0.45 × 0.15 × 0.02 mm

#### Data collection

Bruker SMART APEX diffractometer
16958 measured reflections
4970 independent reflections
3318 reflections with *I* > 2σ(*I*)
*R*_int_ = 0.085

#### Refinement

*R*[*F* ^2^ > 2σ(*F* ^2^)] = 0.055
*wR*(*F* ^2^) = 0.143
*S* = 0.96
4970 reflections
593 parameters
1 restraint
H-atom parameters constrained
Δρ_max_ = 0.18 e Å^−3^
Δρ_min_ = −0.19 e Å^−3^

### 

Data collection: *APEX2* software (Bruker, 2009 ▶); cell refinement: *SAINT* (Bruker, 2009 ▶); data reduction: *SAINT*; program(s) used to solve structure: *SHELXS97* (Sheldrick, 2008 ▶); program(s) used to refine structure: *SHELXL97* (Sheldrick, 2008 ▶); molecular graphics: *X-SEED* (Barbour, 2001 ▶); software used to prepare material for publication: *publCIF* (Westrip, 2010 ▶).

## Supplementary Material

Crystal structure: contains datablocks global, I. DOI: 10.1107/S1600536810029168/jh2187sup1.cif

Structure factors: contains datablocks I. DOI: 10.1107/S1600536810029168/jh2187Isup2.hkl

Additional supplementary materials:  crystallographic information; 3D view; checkCIF report

## Figures and Tables

**Table 1:** Hydrogen-bond geometry (Å, °)

*D*—H⋯*A*	*D*—H	H⋯*A*	*D*⋯*A*	*D*—H⋯*A*
O1—H1⋯O2^i^	0.84	2.11	2.807 (5)	140
O2—H2⋯O3^ii^	0.84	1.97	2.784 (5)	162
O3—H3⋯O4^iii^	0.84	2.00	2.747 (5)	148
O4—H4⋯O1	0.84	1.89	2.722 (4)	173

Symmetry codes: (i) 
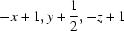
; (ii) 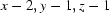; (iii) 
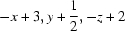
.

**Fig. 1.**
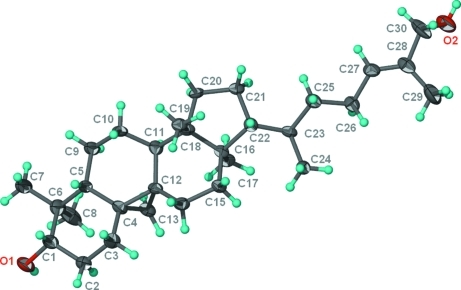
Thermal ellipsoid plot (Barbour, 2001) of one of the two independent molecules of C30H50O2 at the 70% probability level; hydrogen atoms are drawn as spheres of arbitrary radius.

**Fig. 2.**
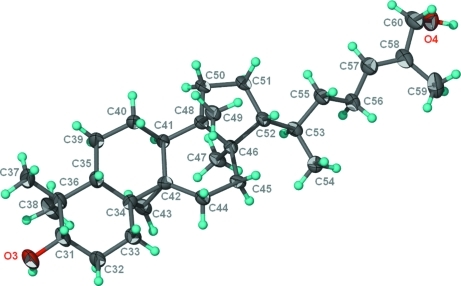
Thermal ellipsoid plot (Barbour, 2001) of the second independent molecule of C30H50O2 at the 70% probability level; hydrogen atoms are drawn as spheres of arbitrary radius.

## supplementary crystallographic information

### Comment

Cycloart-24-ene-3β,26-diol (Scheme I) has been isolated from the leaves of *Garcinia magnostana* (Parveeen *et al.*, 1990), the stem-bark of *Mangifera indica* (Anjaneyulu *et al.*, 1985, 1994) and from the wood of *Schizandar nigra* (Takahashi & Takani, 1975). The structure was elucidated in the studies by spectroscopic methods. The absolute configuration in the present crystal structure analysis is assumed to be that reported in the studies. Cycloart-24-ene-3*β*,26-diol (Fig. 1) in this study was isolated from the leaves of *Aglaia exima*.

### Experimental

The leaves of *Algaia exima* were collected from Kampung Kepayang, Pahang, Malaysia. The leaves (1 kg) were extracted at room temperature with *n*-hexane successively; the insoluble material was removed by filtration after four days. The solvent was evaporated to dryness to give a residue of 25 g; a 15 g portion was subjected to column chromatography over silica gel at a gradient mixture of *n*-hexane and ethyl acetate. Of the 123 fractions, fraction 92 gave a colorless solid, identified by solution NMR, as cycloart-24-ene-3*β*,26-diol. Single crystals were obtained by recrystallization from ethyl acetate.

### Refinement

Carbon-bound H-atoms were placed in calculated positions [C—H 0.95 to 1.00 Å, *U*(H) 1.2 to 1.5*U*(C)] and were included in the refinement in the riding model approximation. The hydroxy H-atoms were similarly placed [O–H 0.84 Å] and their temperature factor similarly tied.

Some 4185 Friedel pairs were merged.

### Crystal data

**Table:**

C_30_H_50_O_2_	*F*(000) = 984
*M**_r_* = 442.70	*D*_x_ = 1.125 Mg m^−^^3^
Monoclinic, *P*2_1_	Mo *K*α radiation, λ = 0.71073 Å
Hall symbol: P 2yb	Cell parameters from 1946 reflections
*a* = 9.9950 (14) Å	θ = 2.3–21.1°
*b* = 7.512 (1) Å	µ = 0.07 mm^−^^1^
*c* = 34.820 (5) Å	*T* = 100 K
β = 91.029 (2)°	Plate, colorless
*V* = 2614.0 (6) Å^3^	0.45 × 0.15 × 0.02 mm
*Z* = 4	

### Data collection

**Table:**

Bruker SMART APEX diffractometer	3318 reflections with *I* > 2σ(*I*)
Radiation source: fine-focus sealed tube	*R*_int_ = 0.085
graphite	θ_max_ = 25.0°, θ_min_ = 1.8°
ω scans	*h* = −11→11
16958 measured reflections	*k* = −8→8
4970 independent reflections	*l* = −36→41

### Refinement

**Table:**

Refinement on *F*^2^	Primary atom site location: structure-invariant direct methods
Least-squares matrix: full	Secondary atom site location: difference Fourier map
*R*[*F*^2^ > 2σ(*F*^2^)] = 0.055	Hydrogen site location: inferred from neighbouring sites
*wR*(*F*^2^) = 0.143	H-atom parameters constrained
*S* = 0.96	*w* = 1/[σ^2^(*F*_o_^2^) + (0.0758*P*)^2^ + 0.1056*P*] where *P* = (*F*_o_^2^ + 2*F*_c_^2^)/3
4970 reflections	(Δ/σ)_max_ = 0.001
593 parameters	Δρ_max_ = 0.18 e Å^−^^3^
1 restraint	Δρ_min_ = −0.19 e Å^−^^3^

### Fractional atomic coordinates and isotropic or equivalent isotropic displacement parameters (Å^2^)

**Table:**

	*x*	*y*	*z*	*U*_iso_*/*U*_eq_	
O1	0.8196 (3)	0.5009 (4)	0.73269 (10)	0.0366 (9)	
H1	0.8842	0.5089	0.7177	0.055*	
O2	−0.0926 (3)	0.0538 (5)	0.28107 (9)	0.0421 (9)	
H2	−0.1424	−0.0187	0.2693	0.063*	
O3	1.7903 (3)	0.7649 (5)	1.24335 (10)	0.0485 (10)	
H3	1.8534	0.7927	1.2289	0.073*	
O4	0.9518 (4)	0.2801 (5)	0.78237 (9)	0.0436 (9)	
H4	0.9051	0.3438	0.7675	0.065*	
C1	0.6990 (5)	0.4641 (6)	0.71105 (14)	0.0298 (12)	
H1a	0.6246	0.4576	0.7298	0.036*	
C2	0.6698 (5)	0.6209 (6)	0.68482 (13)	0.0278 (11)	
H2A	0.7463	0.6387	0.6676	0.033*	
H2B	0.6603	0.7297	0.7006	0.033*	
C3	0.5413 (4)	0.5941 (6)	0.66033 (14)	0.0280 (11)	
H3A	0.4626	0.5951	0.6772	0.034*	
H3B	0.5315	0.6933	0.6418	0.034*	
C4	0.5463 (4)	0.4196 (6)	0.63883 (13)	0.0254 (11)	
C5	0.5667 (5)	0.2676 (6)	0.66768 (13)	0.0267 (11)	
H5	0.4961	0.2843	0.6874	0.032*	
C6	0.7025 (5)	0.2847 (7)	0.68988 (14)	0.0339 (12)	
C7	0.7133 (6)	0.1353 (7)	0.71945 (17)	0.0524 (18)	
H7A	0.7288	0.0220	0.7063	0.079*	
H7B	0.6300	0.1282	0.7338	0.079*	
H7C	0.7881	0.1598	0.7373	0.079*	
C8	0.8233 (4)	0.2766 (7)	0.66345 (15)	0.0433 (14)	
H8A	0.8166	0.1704	0.6472	0.065*	
H8B	0.9058	0.2709	0.6790	0.065*	
H8C	0.8248	0.3832	0.6472	0.065*	
C9	0.5395 (5)	0.0842 (6)	0.64985 (14)	0.0320 (12)	
H9A	0.6117	0.0537	0.6319	0.038*	
H9B	0.5382	−0.0074	0.6703	0.038*	
C10	0.4054 (4)	0.0867 (6)	0.62840 (13)	0.0292 (11)	
H10A	0.3357	0.1359	0.6453	0.035*	
H10B	0.3793	−0.0362	0.6213	0.035*	
C11	0.4152 (4)	0.2002 (6)	0.59238 (13)	0.0238 (11)	
H11	0.4885	0.1462	0.5771	0.029*	
C12	0.4619 (4)	0.3924 (6)	0.60191 (13)	0.0226 (10)	
C13	0.6114 (4)	0.4216 (6)	0.60022 (12)	0.0272 (11)	
H13A	0.6678	0.3184	0.5934	0.033*	
H13B	0.6431	0.5375	0.5904	0.033*	
C14	0.3700 (4)	0.5463 (6)	0.58923 (13)	0.0255 (11)	
H14A	0.4244	0.6562	0.5881	0.031*	
H14B	0.3021	0.5640	0.6092	0.031*	
C15	0.2956 (4)	0.5231 (6)	0.54978 (12)	0.0216 (10)	
H15A	0.2001	0.5536	0.5529	0.026*	
H15B	0.3335	0.6082	0.5312	0.026*	
C16	0.3052 (4)	0.3349 (5)	0.53316 (12)	0.0193 (10)	
C17	0.4415 (4)	0.3143 (6)	0.51311 (13)	0.0233 (10)	
H17A	0.5138	0.3503	0.5308	0.035*	
H17B	0.4427	0.3899	0.4902	0.035*	
H17C	0.4541	0.1897	0.5056	0.035*	
C18	0.2899 (4)	0.1969 (6)	0.56606 (13)	0.0219 (10)	
C19	0.1616 (4)	0.2328 (6)	0.58904 (13)	0.0238 (10)	
H19A	0.1465	0.1341	0.6068	0.036*	
H19B	0.0849	0.2433	0.5712	0.036*	
H19C	0.1721	0.3438	0.6036	0.036*	
C20	0.2706 (4)	0.0201 (5)	0.54461 (12)	0.0224 (10)	
H20A	0.2152	−0.0627	0.5597	0.027*	
H20B	0.3580	−0.0368	0.5398	0.027*	
C21	0.1991 (4)	0.0684 (6)	0.50638 (12)	0.0216 (10)	
H21A	0.1076	0.0181	0.5056	0.026*	
H21B	0.2491	0.0197	0.4845	0.026*	
C22	0.1935 (4)	0.2760 (6)	0.50405 (12)	0.0209 (10)	
H22	0.1060	0.3136	0.5149	0.025*	
C23	0.1965 (4)	0.3444 (5)	0.46270 (13)	0.0221 (10)	
H23	0.2832	0.3056	0.4515	0.027*	
C24	0.1913 (4)	0.5507 (6)	0.46055 (13)	0.0247 (10)	
H24A	0.2726	0.6004	0.4724	0.037*	
H24B	0.1128	0.5938	0.4742	0.037*	
H24C	0.1850	0.5880	0.4336	0.037*	
C25	0.0833 (4)	0.2608 (6)	0.43826 (12)	0.0249 (10)	
H25A	0.0895	0.1296	0.4406	0.030*	
H25B	−0.0035	0.2975	0.4490	0.030*	
C26	0.0835 (4)	0.3098 (6)	0.39569 (12)	0.0256 (11)	
H26A	0.0524	0.4342	0.3926	0.031*	
H26B	0.1763	0.3032	0.3863	0.031*	
C27	−0.0035 (4)	0.1910 (7)	0.37175 (13)	0.0307 (12)	
H27	−0.0161	0.0740	0.3814	0.037*	
C28	−0.0663 (4)	0.2285 (7)	0.33829 (13)	0.0292 (11)	
C29	−0.0642 (6)	0.4038 (7)	0.31926 (16)	0.0541 (17)	
H29A	−0.0183	0.4900	0.3360	0.081*	
H29B	−0.1562	0.4436	0.3141	0.081*	
H29C	−0.0166	0.3942	0.2950	0.081*	
C30	−0.1459 (5)	0.0885 (8)	0.31816 (15)	0.0462 (15)	
H30A	−0.1442	−0.0222	0.3336	0.055*	
H30B	−0.2401	0.1278	0.3154	0.055*	
C31	1.6695 (5)	0.7436 (7)	1.22082 (15)	0.0388 (13)	
H31	1.5927	0.7565	1.2386	0.047*	
C32	1.6570 (5)	0.8907 (6)	1.19104 (14)	0.0338 (12)	
H32A	1.7359	0.8877	1.1743	0.041*	
H32B	1.6562	1.0074	1.2042	0.041*	
C33	1.5287 (5)	0.8723 (6)	1.16604 (14)	0.0314 (12)	
H33A	1.4487	0.8856	1.1822	0.038*	
H33B	1.5262	0.9661	1.1461	0.038*	
C34	1.5290 (5)	0.6896 (6)	1.14735 (14)	0.0297 (12)	
C35	1.5352 (5)	0.5445 (6)	1.17769 (14)	0.0320 (12)	
H35	1.4598	0.5722	1.1952	0.038*	
C36	1.6625 (5)	0.5531 (7)	1.20379 (14)	0.0355 (13)	
C37	1.6508 (6)	0.4222 (7)	1.23772 (16)	0.0543 (17)	
H37A	1.5632	0.4363	1.2495	0.082*	
H37B	1.7215	0.4473	1.2568	0.082*	
H37C	1.6603	0.2999	1.2284	0.082*	
C38	1.7893 (5)	0.5113 (8)	1.18178 (16)	0.0503 (16)	
H38A	1.8032	0.6031	1.1623	0.075*	
H38B	1.7800	0.3949	1.1693	0.075*	
H38C	1.8661	0.5090	1.1997	0.075*	
C39	1.5035 (5)	0.3581 (6)	1.16203 (15)	0.0354 (13)	
H39A	1.5792	0.3149	1.1466	0.043*	
H39B	1.4911	0.2745	1.1837	0.043*	
C40	1.3760 (5)	0.3636 (6)	1.13696 (14)	0.0319 (12)	
H40A	1.3481	0.2408	1.1303	0.038*	
H40B	1.3029	0.4203	1.1514	0.038*	
C41	1.4014 (4)	0.4681 (6)	1.10048 (13)	0.0246 (11)	
H41	1.4767	0.4060	1.0875	0.030*	
C42	1.4523 (4)	0.6587 (6)	1.10937 (14)	0.0268 (11)	
C43	1.6032 (4)	0.6776 (7)	1.10967 (13)	0.0331 (12)	
H43A	1.6412	0.7887	1.0992	0.040*	
H43B	1.6569	0.5692	1.1050	0.040*	
C44	1.3665 (4)	0.8139 (6)	1.09347 (13)	0.0272 (11)	
H44A	1.4231	0.9221	1.0933	0.033*	
H44B	1.2933	0.8358	1.1116	0.033*	
C45	1.3034 (4)	0.7912 (6)	1.05290 (13)	0.0252 (10)	
H45A	1.2085	0.8285	1.0535	0.030*	
H45B	1.3499	0.8711	1.0349	0.030*	
C46	1.3107 (4)	0.5989 (6)	1.03765 (12)	0.0209 (10)	
C47	1.4503 (4)	0.5699 (6)	1.02068 (13)	0.0263 (11)	
H47A	1.5190	0.6088	1.0393	0.039*	
H47B	1.4580	0.6391	0.9970	0.039*	
H47C	1.4627	0.4432	1.0150	0.039*	
C48	1.2828 (4)	0.4665 (6)	1.07123 (13)	0.0245 (11)	
C49	1.1492 (4)	0.5086 (6)	1.09087 (13)	0.0280 (11)	
H49A	1.1295	0.4152	1.1096	0.042*	
H49B	1.0771	0.5138	1.0714	0.042*	
H49C	1.1562	0.6236	1.1040	0.042*	
C50	1.2662 (4)	0.2873 (6)	1.05027 (13)	0.0260 (11)	
H50A	1.2061	0.2073	1.0646	0.031*	
H50B	1.3539	0.2278	1.0474	0.031*	
C51	1.2046 (4)	0.3346 (6)	1.01051 (14)	0.0282 (11)	
H51A	1.2587	0.2819	0.9899	0.034*	
H51B	1.1124	0.2872	1.0082	0.034*	
C52	1.2033 (4)	0.5400 (6)	1.00700 (13)	0.0239 (10)	
H52	1.1146	0.5814	1.0164	0.029*	
C53	1.2148 (4)	0.6056 (6)	0.96550 (13)	0.0248 (11)	
H53	1.3036	0.5663	0.9558	0.030*	
C54	1.2084 (5)	0.8091 (6)	0.96208 (14)	0.0322 (12)	
H54A	1.2865	0.8616	0.9752	0.048*	
H54B	1.1265	0.8526	0.9740	0.048*	
H54C	1.2083	0.8430	0.9349	0.048*	
C55	1.1061 (4)	0.5187 (6)	0.94001 (12)	0.0289 (11)	
H55A	1.1120	0.3879	0.9431	0.035*	
H55B	1.0174	0.5565	0.9493	0.035*	
C56	1.1143 (4)	0.5629 (7)	0.89736 (12)	0.0287 (11)	
H56A	1.0759	0.6826	0.8927	0.034*	
H56B	1.2094	0.5658	0.8899	0.034*	
C57	1.0402 (5)	0.4284 (7)	0.87294 (13)	0.0347 (12)	
H57	1.0464	0.3083	0.8814	0.042*	
C58	0.9668 (4)	0.4553 (7)	0.84101 (13)	0.0341 (12)	
C59	0.9430 (6)	0.6328 (8)	0.82220 (16)	0.0490 (16)	
H59A	0.9726	0.7280	0.8396	0.074*	
H59B	0.8474	0.6469	0.8163	0.074*	
H59C	0.9936	0.6394	0.7984	0.074*	
C60	0.9049 (5)	0.2981 (8)	0.82061 (14)	0.0441 (14)	
H60A	0.9263	0.1882	0.8352	0.053*	
H60B	0.8064	0.3121	0.8199	0.053*	

### Atomic displacement parameters (Å^2^)

**Table:**

	*U*^11^	*U*^22^	*U*^33^	*U*^12^	*U*^13^	*U*^23^
O1	0.0353 (19)	0.030 (2)	0.044 (2)	−0.0015 (16)	−0.0179 (17)	−0.0067 (17)
O2	0.048 (2)	0.046 (2)	0.033 (2)	−0.0128 (18)	0.0014 (17)	−0.0135 (18)
O3	0.050 (2)	0.044 (2)	0.050 (2)	−0.001 (2)	−0.0276 (19)	−0.009 (2)
O4	0.061 (2)	0.041 (2)	0.028 (2)	0.0001 (19)	−0.0149 (17)	0.0036 (17)
C1	0.026 (3)	0.026 (3)	0.037 (3)	−0.003 (2)	−0.011 (2)	0.000 (2)
C2	0.031 (3)	0.024 (3)	0.029 (3)	−0.004 (2)	−0.003 (2)	−0.002 (2)
C3	0.024 (2)	0.023 (3)	0.037 (3)	0.004 (2)	−0.005 (2)	0.000 (2)
C4	0.023 (2)	0.022 (3)	0.030 (3)	0.001 (2)	−0.008 (2)	−0.006 (2)
C5	0.035 (3)	0.015 (2)	0.029 (3)	0.000 (2)	−0.011 (2)	−0.001 (2)
C6	0.033 (3)	0.028 (3)	0.040 (3)	0.002 (2)	−0.014 (2)	0.002 (2)
C7	0.071 (4)	0.024 (3)	0.061 (4)	−0.008 (3)	−0.042 (3)	0.007 (3)
C8	0.029 (3)	0.043 (3)	0.057 (3)	0.013 (2)	−0.018 (2)	−0.021 (3)
C9	0.038 (3)	0.019 (3)	0.038 (3)	−0.001 (2)	−0.015 (2)	0.000 (2)
C10	0.030 (2)	0.017 (2)	0.040 (3)	−0.007 (2)	−0.014 (2)	0.002 (2)
C11	0.021 (2)	0.023 (3)	0.028 (3)	0.0027 (19)	−0.006 (2)	−0.001 (2)
C12	0.020 (2)	0.020 (2)	0.028 (3)	−0.0056 (18)	−0.005 (2)	0.000 (2)
C13	0.023 (2)	0.029 (3)	0.030 (3)	−0.005 (2)	−0.004 (2)	−0.005 (2)
C14	0.023 (2)	0.018 (2)	0.035 (3)	0.0005 (19)	−0.003 (2)	−0.001 (2)
C15	0.022 (2)	0.018 (2)	0.025 (3)	0.0038 (18)	−0.001 (2)	−0.006 (2)
C16	0.010 (2)	0.022 (2)	0.026 (3)	0.0001 (17)	0.0004 (18)	−0.003 (2)
C17	0.016 (2)	0.022 (2)	0.032 (3)	−0.0010 (19)	0.0014 (19)	−0.001 (2)
C18	0.017 (2)	0.017 (2)	0.032 (3)	−0.0007 (18)	−0.0018 (19)	0.000 (2)
C19	0.020 (2)	0.023 (3)	0.028 (3)	−0.0021 (18)	−0.0008 (19)	−0.001 (2)
C20	0.022 (2)	0.014 (2)	0.031 (3)	−0.0048 (18)	0.000 (2)	0.003 (2)
C21	0.017 (2)	0.020 (2)	0.027 (3)	−0.0037 (19)	0.0011 (19)	−0.002 (2)
C22	0.017 (2)	0.024 (2)	0.022 (2)	0.0003 (19)	0.0012 (18)	−0.001 (2)
C23	0.017 (2)	0.019 (2)	0.030 (3)	−0.0030 (18)	−0.0026 (19)	−0.005 (2)
C24	0.022 (2)	0.026 (3)	0.027 (3)	−0.004 (2)	−0.005 (2)	0.002 (2)
C25	0.020 (2)	0.028 (3)	0.027 (3)	−0.0027 (19)	0.0003 (19)	−0.002 (2)
C26	0.017 (2)	0.032 (3)	0.028 (3)	−0.001 (2)	−0.0001 (19)	−0.002 (2)
C27	0.034 (3)	0.033 (3)	0.025 (3)	−0.016 (2)	0.005 (2)	−0.001 (2)
C28	0.022 (2)	0.038 (3)	0.028 (3)	−0.006 (2)	0.004 (2)	−0.010 (2)
C29	0.069 (4)	0.048 (4)	0.045 (4)	0.003 (3)	−0.031 (3)	−0.009 (3)
C30	0.036 (3)	0.071 (4)	0.031 (3)	−0.022 (3)	−0.003 (2)	−0.017 (3)
C31	0.036 (3)	0.042 (3)	0.038 (3)	0.002 (2)	−0.018 (2)	−0.008 (3)
C32	0.039 (3)	0.025 (3)	0.037 (3)	0.001 (2)	−0.013 (2)	−0.006 (2)
C33	0.030 (3)	0.027 (3)	0.037 (3)	0.003 (2)	−0.007 (2)	−0.005 (2)
C34	0.033 (3)	0.021 (3)	0.035 (3)	0.003 (2)	−0.010 (2)	−0.003 (2)
C35	0.036 (3)	0.024 (3)	0.036 (3)	0.006 (2)	−0.014 (2)	−0.005 (2)
C36	0.038 (3)	0.027 (3)	0.040 (3)	0.004 (2)	−0.020 (2)	−0.007 (2)
C37	0.077 (4)	0.031 (3)	0.053 (4)	0.000 (3)	−0.040 (3)	0.001 (3)
C38	0.039 (3)	0.047 (4)	0.063 (4)	0.013 (3)	−0.024 (3)	−0.025 (3)
C39	0.045 (3)	0.021 (3)	0.039 (3)	0.002 (2)	−0.018 (2)	−0.004 (2)
C40	0.038 (3)	0.021 (3)	0.037 (3)	−0.003 (2)	−0.014 (2)	0.000 (2)
C41	0.024 (2)	0.018 (2)	0.032 (3)	0.0019 (19)	−0.008 (2)	0.000 (2)
C42	0.024 (2)	0.020 (2)	0.036 (3)	−0.0025 (19)	−0.005 (2)	0.001 (2)
C43	0.025 (2)	0.034 (3)	0.040 (3)	−0.002 (2)	−0.003 (2)	−0.005 (2)
C44	0.023 (2)	0.025 (3)	0.034 (3)	−0.001 (2)	−0.005 (2)	−0.002 (2)
C45	0.022 (2)	0.022 (2)	0.031 (3)	−0.002 (2)	−0.003 (2)	−0.006 (2)
C46	0.018 (2)	0.025 (3)	0.020 (2)	−0.0017 (19)	0.0013 (18)	0.000 (2)
C47	0.016 (2)	0.029 (3)	0.034 (3)	0.001 (2)	0.0000 (19)	−0.009 (2)
C48	0.020 (2)	0.022 (3)	0.031 (3)	−0.0010 (19)	−0.004 (2)	0.000 (2)
C49	0.022 (2)	0.031 (3)	0.031 (3)	−0.0029 (19)	−0.001 (2)	0.000 (2)
C50	0.022 (2)	0.025 (3)	0.030 (3)	−0.005 (2)	−0.005 (2)	−0.003 (2)
C51	0.021 (2)	0.029 (3)	0.034 (3)	−0.005 (2)	−0.006 (2)	0.000 (2)
C52	0.017 (2)	0.024 (3)	0.030 (3)	0.0017 (19)	−0.002 (2)	−0.003 (2)
C53	0.016 (2)	0.030 (3)	0.028 (3)	−0.0027 (19)	0.000 (2)	−0.002 (2)
C54	0.032 (3)	0.033 (3)	0.031 (3)	−0.004 (2)	−0.005 (2)	0.006 (2)
C55	0.025 (2)	0.035 (3)	0.027 (3)	−0.005 (2)	−0.003 (2)	0.000 (2)
C56	0.026 (2)	0.037 (3)	0.023 (3)	−0.001 (2)	−0.003 (2)	−0.001 (2)
C57	0.034 (3)	0.042 (3)	0.028 (3)	−0.009 (2)	−0.001 (2)	0.005 (2)
C58	0.025 (2)	0.052 (3)	0.025 (3)	−0.009 (2)	0.003 (2)	0.005 (3)
C59	0.053 (3)	0.056 (4)	0.038 (3)	0.018 (3)	−0.014 (3)	−0.006 (3)
C60	0.042 (3)	0.057 (4)	0.032 (3)	−0.021 (3)	−0.008 (2)	0.009 (3)

### Geometric parameters (Å, °)

**Table:**

O1—C1	1.437 (5)	C29—H29B	0.9800
O1—H1	0.8400	C29—H29C	0.9800
O2—C30	1.430 (6)	C30—H30A	0.9900
O2—H2	0.8400	C30—H30B	0.9900
O3—C31	1.437 (5)	C31—C32	1.519 (7)
O3—H3	0.8400	C31—C36	1.550 (7)
O4—C60	1.426 (6)	C31—H31	1.0000
O4—H4	0.8400	C32—C33	1.543 (6)
C1—C2	1.516 (6)	C32—H32A	0.9900
C1—C6	1.537 (7)	C32—H32B	0.9900
C1—H1a	1.0000	C33—C34	1.519 (6)
C2—C3	1.542 (6)	C33—H33A	0.9900
C2—H2A	0.9900	C33—H33B	0.9900
C2—H2B	0.9900	C34—C35	1.518 (7)
C3—C4	1.510 (6)	C34—C43	1.521 (7)
C3—H3A	0.9900	C34—C42	1.534 (6)
C3—H3B	0.9900	C35—C39	1.534 (6)
C4—C13	1.504 (6)	C35—C36	1.551 (6)
C4—C5	1.532 (6)	C35—H35	1.0000
C4—C12	1.538 (6)	C36—C38	1.525 (7)
C5—C9	1.534 (6)	C36—C37	1.543 (7)
C5—C6	1.555 (6)	C37—H37A	0.9800
C5—H5	1.0000	C37—H37B	0.9800
C6—C8	1.533 (7)	C37—H37C	0.9800
C6—C7	1.525 (7)	C38—H38A	0.9800
C7—H7A	0.9800	C38—H38B	0.9800
C7—H7B	0.9800	C38—H38C	0.9800
C7—H7C	0.9800	C39—C40	1.532 (6)
C8—H8A	0.9800	C39—H39A	0.9900
C8—H8B	0.9800	C39—H39B	0.9900
C8—H8C	0.9800	C40—C41	1.519 (6)
C9—C10	1.523 (6)	C40—H40A	0.9900
C9—H9A	0.9900	C40—H40B	0.9900
C9—H9B	0.9900	C41—C48	1.549 (5)
C10—C11	1.521 (6)	C41—C42	1.549 (6)
C10—H10A	0.9900	C41—H41	1.0000
C10—H10B	0.9900	C42—C43	1.514 (6)
C11—C18	1.539 (5)	C42—C44	1.544 (6)
C11—C12	1.552 (6)	C43—H43A	0.9900
C11—H11	1.0000	C43—H43B	0.9900
C12—C13	1.513 (6)	C44—C45	1.546 (6)
C12—C14	1.536 (6)	C44—H44A	0.9900
C13—H13A	0.9900	C44—H44B	0.9900
C13—H13B	0.9900	C45—C46	1.541 (6)
C14—C15	1.560 (6)	C45—H45A	0.9900
C14—H14A	0.9900	C45—H45B	0.9900
C14—H14B	0.9900	C46—C47	1.541 (6)
C15—C16	1.531 (6)	C46—C52	1.564 (6)
C15—H15A	0.9900	C46—C48	1.564 (6)
C15—H15B	0.9900	C47—H47A	0.9800
C16—C17	1.550 (6)	C47—H47B	0.9800
C16—C18	1.554 (6)	C47—H47C	0.9800
C16—C22	1.558 (5)	C48—C50	1.539 (6)
C17—H17A	0.9800	C48—C49	1.543 (6)
C17—H17B	0.9800	C49—H49A	0.9800
C17—H17C	0.9800	C49—H49B	0.9800
C18—C20	1.534 (6)	C49—H49C	0.9800
C18—C19	1.548 (6)	C50—C51	1.546 (6)
C19—H19A	0.9800	C50—H50A	0.9900
C19—H19B	0.9800	C50—H50B	0.9900
C19—H19C	0.9800	C51—C52	1.548 (6)
C20—C21	1.542 (6)	C51—H51A	0.9900
C20—H20A	0.9900	C51—H51B	0.9900
C20—H20B	0.9900	C52—C53	1.533 (6)
C21—C22	1.563 (6)	C52—H52	1.0000
C21—H21A	0.9900	C53—C54	1.535 (6)
C21—H21B	0.9900	C53—C55	1.536 (6)
C22—C23	1.530 (6)	C53—H53	1.0000
C22—H22	1.0000	C54—H54A	0.9800
C23—C25	1.538 (5)	C54—H54B	0.9800
C23—C24	1.552 (6)	C54—H54C	0.9800
C23—H23	1.0000	C55—C56	1.526 (6)
C24—H24A	0.9800	C55—H55A	0.9900
C24—H24B	0.9800	C55—H55B	0.9900
C24—H24C	0.9800	C56—C57	1.507 (6)
C25—C26	1.528 (6)	C56—H56A	0.9900
C25—H25A	0.9900	C56—H56B	0.9900
C25—H25B	0.9900	C57—C58	1.336 (6)
C26—C27	1.490 (6)	C57—H57	0.9500
C26—H26A	0.9900	C58—C59	1.503 (7)
C26—H26B	0.9900	C58—C60	1.506 (7)
C27—C28	1.344 (6)	C59—H59A	0.9800
C27—H27	0.9500	C59—H59B	0.9800
C28—C29	1.474 (7)	C59—H59C	0.9800
C28—C30	1.486 (7)	C60—H60A	0.9900
C29—H29A	0.9800	C60—H60B	0.9900
			
C1—O1—H1	109.5	C28—C30—H30B	109.6
C30—O2—H2	109.5	H30A—C30—H30B	108.1
C31—O3—H3	109.5	O3—C31—C32	110.5 (4)
C60—O4—H4	109.5	O3—C31—C36	110.1 (4)
O1—C1—C2	108.5 (4)	C32—C31—C36	114.1 (4)
O1—C1—C6	113.2 (4)	O3—C31—H31	107.3
C2—C1—C6	113.5 (4)	C32—C31—H31	107.3
O1—C1—H1a	107.1	C36—C31—H31	107.3
C2—C1—H1a	107.1	C31—C32—C33	112.1 (4)
C6—C1—H1a	107.1	C31—C32—H32A	109.2
C1—C2—C3	112.4 (4)	C33—C32—H32A	109.2
C1—C2—H2A	109.1	C31—C32—H32B	109.2
C3—C2—H2A	109.1	C33—C32—H32B	109.2
C1—C2—H2B	109.1	H32A—C32—H32B	107.9
C3—C2—H2B	109.1	C34—C33—C32	108.4 (4)
H2A—C2—H2B	107.8	C34—C33—H33A	110.0
C4—C3—C2	110.7 (4)	C32—C33—H33A	110.0
C4—C3—H3A	109.5	C34—C33—H33B	110.0
C2—C3—H3A	109.5	C32—C33—H33B	110.0
C4—C3—H3B	109.5	H33A—C33—H33B	108.4
C2—C3—H3B	109.5	C35—C34—C33	110.5 (4)
H3A—C3—H3B	108.1	C35—C34—C43	122.9 (4)
C13—C4—C3	116.9 (4)	C33—C34—C43	115.4 (4)
C13—C4—C5	122.6 (4)	C35—C34—C42	120.3 (4)
C3—C4—C5	109.1 (4)	C33—C34—C42	120.1 (4)
C13—C4—C12	59.6 (3)	C43—C34—C42	59.4 (3)
C3—C4—C12	120.5 (4)	C34—C35—C39	113.7 (4)
C5—C4—C12	120.9 (4)	C34—C35—C36	113.6 (4)
C4—C5—C9	112.6 (4)	C39—C35—C36	114.1 (4)
C4—C5—C6	111.7 (4)	C34—C35—H35	104.7
C9—C5—C6	115.0 (4)	C39—C35—H35	104.7
C4—C5—H5	105.6	C36—C35—H35	104.7
C9—C5—H5	105.6	C38—C36—C37	109.2 (4)
C6—C5—H5	105.6	C38—C36—C31	110.5 (4)
C8—C6—C7	109.2 (4)	C37—C36—C31	107.4 (4)
C8—C6—C1	110.3 (4)	C38—C36—C35	112.2 (4)
C7—C6—C1	108.9 (4)	C37—C36—C35	110.5 (4)
C8—C6—C5	112.9 (4)	C31—C36—C35	107.0 (4)
C7—C6—C5	109.0 (4)	C36—C37—H37A	109.5
C1—C6—C5	106.5 (4)	C36—C37—H37B	109.5
C6—C7—H7A	109.5	H37A—C37—H37B	109.5
C6—C7—H7B	109.5	C36—C37—H37C	109.5
H7A—C7—H7B	109.5	H37A—C37—H37C	109.5
C6—C7—H7C	109.5	H37B—C37—H37C	109.5
H7A—C7—H7C	109.5	C36—C38—H38A	109.5
H7B—C7—H7C	109.5	C36—C38—H38B	109.5
C6—C8—H8A	109.5	H38A—C38—H38B	109.5
C6—C8—H8B	109.5	C36—C38—H38C	109.5
H8A—C8—H8B	109.5	H38A—C38—H38C	109.5
C6—C8—H8C	109.5	H38B—C38—H38C	109.5
H8A—C8—H8C	109.5	C40—C39—C35	110.0 (4)
H8B—C8—H8C	109.5	C40—C39—H39A	109.7
C10—C9—C5	109.6 (4)	C35—C39—H39A	109.7
C10—C9—H9A	109.8	C40—C39—H39B	109.7
C5—C9—H9A	109.8	C35—C39—H39B	109.7
C10—C9—H9B	109.8	H39A—C39—H39B	108.2
C5—C9—H9B	109.8	C41—C40—C39	109.9 (4)
H9A—C9—H9B	108.2	C41—C40—H40A	109.7
C9—C10—C11	110.0 (4)	C39—C40—H40A	109.7
C9—C10—H10A	109.7	C41—C40—H40B	109.7
C11—C10—H10A	109.7	C39—C40—H40B	109.7
C9—C10—H10B	109.7	H40A—C40—H40B	108.2
C11—C10—H10B	109.7	C40—C41—C48	114.1 (4)
H10A—C10—H10B	108.2	C40—C41—C42	111.7 (4)
C10—C11—C18	114.7 (4)	C48—C41—C42	112.6 (3)
C10—C11—C12	111.6 (4)	C40—C41—H41	105.9
C18—C11—C12	112.4 (3)	C48—C41—H41	105.9
C10—C11—H11	105.8	C42—C41—H41	105.9
C18—C11—H11	105.8	C43—C42—C34	59.9 (3)
C12—C11—H11	105.8	C43—C42—C44	118.6 (4)
C13—C12—C14	117.8 (4)	C34—C42—C44	117.4 (4)
C13—C12—C4	59.1 (3)	C43—C42—C41	114.3 (4)
C14—C12—C4	117.1 (4)	C34—C42—C41	118.0 (4)
C13—C12—C11	114.8 (4)	C44—C42—C41	116.6 (3)
C14—C12—C11	117.6 (3)	C42—C43—C34	60.7 (3)
C4—C12—C11	117.4 (4)	C42—C43—H43A	117.7
C4—C13—C12	61.3 (3)	C34—C43—H43A	117.7
C4—C13—H13A	117.6	C42—C43—H43B	117.7
C12—C13—H13A	117.6	C34—C43—H43B	117.7
C4—C13—H13B	117.6	H43A—C43—H43B	114.8
C12—C13—H13B	117.6	C42—C44—C45	117.4 (4)
H13A—C13—H13B	114.7	C42—C44—H44A	108.0
C12—C14—C15	116.2 (4)	C45—C44—H44A	108.0
C12—C14—H14A	108.2	C42—C44—H44B	108.0
C15—C14—H14A	108.2	C45—C44—H44B	108.0
C12—C14—H14B	108.2	H44A—C44—H44B	107.2
C15—C14—H14B	108.2	C46—C45—C44	113.4 (4)
H14A—C14—H14B	107.4	C46—C45—H45A	108.9
C16—C15—C14	113.8 (3)	C44—C45—H45A	108.9
C16—C15—H15A	108.8	C46—C45—H45B	108.9
C14—C15—H15A	108.8	C44—C45—H45B	108.9
C16—C15—H15B	108.8	H45A—C45—H45B	107.7
C14—C15—H15B	108.8	C47—C46—C45	108.3 (3)
H15A—C15—H15B	107.7	C47—C46—C52	108.3 (3)
C15—C16—C17	108.9 (3)	C45—C46—C52	117.7 (3)
C15—C16—C18	109.3 (3)	C47—C46—C48	111.8 (3)
C17—C16—C18	111.4 (3)	C45—C46—C48	109.2 (4)
C15—C16—C22	117.3 (3)	C52—C46—C48	101.6 (3)
C17—C16—C22	107.7 (3)	C46—C47—H47A	109.5
C18—C16—C22	102.2 (3)	C46—C47—H47B	109.5
C16—C17—H17A	109.5	H47A—C47—H47B	109.5
C16—C17—H17B	109.5	C46—C47—H47C	109.5
H17A—C17—H17B	109.5	H47A—C47—H47C	109.5
C16—C17—H17C	109.5	H47B—C47—H47C	109.5
H17A—C17—H17C	109.5	C50—C48—C49	107.6 (4)
H17B—C17—H17C	109.5	C50—C48—C41	113.2 (4)
C20—C18—C11	113.5 (3)	C49—C48—C41	111.4 (4)
C20—C18—C19	107.7 (3)	C50—C48—C46	102.8 (3)
C11—C18—C19	111.2 (4)	C49—C48—C46	111.6 (3)
C20—C18—C16	103.4 (3)	C41—C48—C46	110.0 (3)
C11—C18—C16	109.8 (3)	C48—C49—H49A	109.5
C19—C18—C16	111.0 (3)	C48—C49—H49B	109.5
C18—C19—H19A	109.5	H49A—C49—H49B	109.5
C18—C19—H19B	109.5	C48—C49—H49C	109.5
H19A—C19—H19B	109.5	H49A—C49—H49C	109.5
C18—C19—H19C	109.5	H49B—C49—H49C	109.5
H19A—C19—H19C	109.5	C48—C50—C51	105.1 (4)
H19B—C19—H19C	109.5	C48—C50—H50A	110.7
C18—C20—C21	105.6 (3)	C51—C50—H50A	110.7
C18—C20—H20A	110.6	C48—C50—H50B	110.7
C21—C20—H20A	110.6	C51—C50—H50B	110.7
C18—C20—H20B	110.6	H50A—C50—H50B	108.8
C21—C20—H20B	110.6	C52—C51—C50	107.6 (4)
H20A—C20—H20B	108.7	C52—C51—H51A	110.2
C20—C21—C22	107.2 (3)	C50—C51—H51A	110.2
C20—C21—H21A	110.3	C52—C51—H51B	110.2
C22—C21—H21A	110.3	C50—C51—H51B	110.2
C20—C21—H21B	110.3	H51A—C51—H51B	108.5
C22—C21—H21B	110.3	C53—C52—C51	113.2 (4)
H21A—C21—H21B	108.5	C53—C52—C46	119.4 (4)
C23—C22—C16	119.4 (4)	C51—C52—C46	102.9 (3)
C23—C22—C21	112.5 (4)	C53—C52—H52	106.9
C16—C22—C21	103.0 (3)	C51—C52—H52	106.9
C23—C22—H22	107.1	C46—C52—H52	106.9
C16—C22—H22	107.1	C54—C53—C55	110.5 (4)
C21—C22—H22	107.1	C54—C53—C52	112.9 (4)
C22—C23—C25	110.9 (3)	C55—C53—C52	110.1 (4)
C22—C23—C24	112.3 (4)	C54—C53—H53	107.7
C25—C23—C24	110.9 (4)	C55—C53—H53	107.7
C22—C23—H23	107.5	C52—C53—H53	107.7
C25—C23—H23	107.5	C53—C54—H54A	109.5
C24—C23—H23	107.5	C53—C54—H54B	109.5
C23—C24—H24A	109.5	H54A—C54—H54B	109.5
C23—C24—H24B	109.5	C53—C54—H54C	109.5
H24A—C24—H24B	109.5	H54A—C54—H54C	109.5
C23—C24—H24C	109.5	H54B—C54—H54C	109.5
H24A—C24—H24C	109.5	C56—C55—C53	114.8 (4)
H24B—C24—H24C	109.5	C56—C55—H55A	108.6
C26—C25—C23	115.1 (4)	C53—C55—H55A	108.6
C26—C25—H25A	108.5	C56—C55—H55B	108.6
C23—C25—H25A	108.5	C53—C55—H55B	108.6
C26—C25—H25B	108.5	H55A—C55—H55B	107.5
C23—C25—H25B	108.5	C57—C56—C55	111.6 (4)
H25A—C25—H25B	107.5	C57—C56—H56A	109.3
C27—C26—C25	112.8 (4)	C55—C56—H56A	109.3
C27—C26—H26A	109.0	C57—C56—H56B	109.3
C25—C26—H26A	109.0	C55—C56—H56B	109.3
C27—C26—H26B	109.0	H56A—C56—H56B	108.0
C25—C26—H26B	109.0	C58—C57—C56	128.7 (5)
H26A—C26—H26B	107.8	C58—C57—H57	115.7
C28—C27—C26	128.2 (4)	C56—C57—H57	115.7
C28—C27—H27	115.9	C57—C58—C59	125.3 (5)
C26—C27—H27	115.9	C57—C58—C60	119.2 (5)
C27—C28—C29	124.5 (4)	C59—C58—C60	115.5 (4)
C27—C28—C30	119.9 (5)	C58—C59—H59A	109.5
C29—C28—C30	115.6 (4)	C58—C59—H59B	109.5
C28—C29—H29A	109.5	H59A—C59—H59B	109.5
C28—C29—H29B	109.5	C58—C59—H59C	109.5
H29A—C29—H29B	109.5	H59A—C59—H59C	109.5
C28—C29—H29C	109.5	H59B—C59—H59C	109.5
H29A—C29—H29C	109.5	O4—C60—C58	112.1 (4)
H29B—C29—H29C	109.5	O4—C60—H60A	109.2
O2—C30—C28	110.5 (4)	C58—C60—H60A	109.2
O2—C30—H30A	109.6	O4—C60—H60B	109.2
C28—C30—H30A	109.6	C58—C60—H60B	109.2
O2—C30—H30B	109.6	H60A—C60—H60B	107.9
			
O1—C1—C2—C3	−179.9 (4)	O3—C31—C32—C33	179.7 (4)
C6—C1—C2—C3	53.4 (5)	C36—C31—C32—C33	55.1 (6)
C1—C2—C3—C4	−53.2 (5)	C31—C32—C33—C34	−56.6 (6)
C2—C3—C4—C13	−87.5 (5)	C32—C33—C34—C35	58.7 (5)
C2—C3—C4—C5	57.1 (5)	C32—C33—C34—C43	−86.3 (5)
C2—C3—C4—C12	−156.4 (4)	C32—C33—C34—C42	−154.2 (4)
C13—C4—C5—C9	−51.6 (5)	C33—C34—C35—C39	166.6 (4)
C3—C4—C5—C9	166.2 (4)	C43—C34—C35—C39	−51.5 (6)
C12—C4—C5—C9	19.9 (6)	C42—C34—C35—C39	19.6 (6)
C13—C4—C5—C6	79.5 (5)	C33—C34—C35—C36	−60.6 (5)
C3—C4—C5—C6	−62.7 (5)	C43—C34—C35—C36	81.2 (5)
C12—C4—C5—C6	151.0 (4)	C42—C34—C35—C36	152.4 (4)
O1—C1—C6—C8	−56.3 (5)	O3—C31—C36—C38	−53.7 (5)
C2—C1—C6—C8	67.9 (5)	C32—C31—C36—C38	71.1 (5)
O1—C1—C6—C7	63.5 (5)	O3—C31—C36—C37	65.3 (5)
C2—C1—C6—C7	−172.3 (4)	C32—C31—C36—C37	−169.9 (5)
O1—C1—C6—C5	−179.1 (4)	O3—C31—C36—C35	−176.1 (4)
C2—C1—C6—C5	−54.9 (5)	C32—C31—C36—C35	−51.3 (6)
C4—C5—C6—C8	−61.4 (5)	C34—C35—C36—C38	−67.2 (5)
C9—C5—C6—C8	68.4 (6)	C39—C35—C36—C38	65.4 (6)
C4—C5—C6—C7	177.1 (4)	C34—C35—C36—C37	170.8 (4)
C9—C5—C6—C7	−53.1 (6)	C39—C35—C36—C37	−56.7 (6)
C4—C5—C6—C1	59.8 (5)	C34—C35—C36—C31	54.2 (6)
C9—C5—C6—C1	−170.3 (4)	C39—C35—C36—C31	−173.3 (5)
C4—C5—C9—C10	−49.5 (5)	C34—C35—C39—C40	−47.9 (6)
C6—C5—C9—C10	−178.9 (4)	C36—C35—C39—C40	179.6 (4)
C5—C9—C10—C11	70.3 (5)	C35—C39—C40—C41	68.2 (5)
C9—C10—C11—C18	173.8 (4)	C39—C40—C41—C48	174.4 (4)
C9—C10—C11—C12	−56.8 (5)	C39—C40—C41—C42	−56.5 (5)
C3—C4—C12—C13	105.3 (5)	C35—C34—C42—C43	−112.7 (5)
C5—C4—C12—C13	−112.1 (5)	C33—C34—C42—C43	103.4 (5)
C13—C4—C12—C14	−107.6 (4)	C35—C34—C42—C44	138.6 (4)
C3—C4—C12—C14	−2.3 (6)	C33—C34—C42—C44	−5.3 (7)
C5—C4—C12—C14	140.2 (4)	C43—C34—C42—C44	−108.8 (5)
C13—C4—C12—C11	103.9 (5)	C35—C34—C42—C41	−9.3 (6)
C3—C4—C12—C11	−150.8 (4)	C33—C34—C42—C41	−153.2 (4)
C5—C4—C12—C11	−8.2 (6)	C43—C34—C42—C41	103.3 (5)
C10—C11—C12—C13	92.5 (5)	C40—C41—C42—C43	94.8 (5)
C18—C11—C12—C13	−137.0 (4)	C48—C41—C42—C43	−135.2 (4)
C10—C11—C12—C14	−122.3 (4)	C40—C41—C42—C34	27.4 (6)
C18—C11—C12—C14	8.2 (6)	C48—C41—C42—C34	157.3 (4)
C10—C11—C12—C4	26.0 (6)	C40—C41—C42—C44	−120.8 (4)
C18—C11—C12—C4	156.5 (4)	C48—C41—C42—C44	9.2 (6)
C3—C4—C13—C12	−111.2 (4)	C44—C42—C43—C34	106.9 (5)
C5—C4—C13—C12	109.4 (4)	C41—C42—C43—C34	−109.5 (4)
C14—C12—C13—C4	106.6 (4)	C35—C34—C43—C42	108.4 (5)
C11—C12—C13—C4	−108.3 (4)	C33—C34—C43—C42	−111.4 (4)
C13—C12—C14—C15	106.9 (4)	C43—C42—C44—C45	103.8 (5)
C4—C12—C14—C15	174.4 (4)	C34—C42—C44—C45	172.7 (4)
C11—C12—C14—C15	−37.2 (6)	C41—C42—C44—C45	−39.0 (6)
C12—C14—C15—C16	10.9 (5)	C42—C44—C45—C46	12.5 (6)
C14—C15—C16—C17	−81.3 (4)	C44—C45—C46—C47	−82.4 (4)
C14—C15—C16—C18	40.5 (5)	C44—C45—C46—C52	154.6 (4)
C14—C15—C16—C22	156.2 (4)	C44—C45—C46—C48	39.6 (5)
C10—C11—C18—C20	−71.9 (5)	C40—C41—C48—C50	−73.8 (5)
C12—C11—C18—C20	159.2 (4)	C42—C41—C48—C50	157.5 (4)
C10—C11—C18—C19	49.8 (5)	C40—C41—C48—C49	47.7 (5)
C12—C11—C18—C19	−79.2 (5)	C42—C41—C48—C49	−81.0 (5)
C10—C11—C18—C16	173.0 (4)	C40—C41—C48—C46	171.9 (4)
C12—C11—C18—C16	44.1 (5)	C42—C41—C48—C46	43.3 (5)
C15—C16—C18—C20	168.1 (3)	C47—C46—C48—C50	−70.8 (4)
C17—C16—C18—C20	−71.6 (4)	C45—C46—C48—C50	169.4 (3)
C22—C16—C18—C20	43.1 (4)	C52—C46—C48—C50	44.4 (4)
C15—C16—C18—C11	−70.5 (4)	C47—C46—C48—C49	174.1 (3)
C17—C16—C18—C11	49.8 (5)	C45—C46—C48—C49	54.3 (4)
C22—C16—C18—C11	164.5 (3)	C52—C46—C48—C49	−70.6 (4)
C15—C16—C18—C19	52.9 (4)	C47—C46—C48—C41	49.9 (5)
C17—C16—C18—C19	173.2 (3)	C45—C46—C48—C41	−69.8 (4)
C22—C16—C18—C19	−72.1 (4)	C52—C46—C48—C41	165.2 (4)
C11—C18—C20—C21	−149.6 (4)	C49—C48—C50—C51	86.1 (4)
C19—C18—C20—C21	86.8 (4)	C41—C48—C50—C51	−150.4 (4)
C16—C18—C20—C21	−30.8 (4)	C46—C48—C50—C51	−31.8 (4)
C18—C20—C21—C22	6.7 (4)	C48—C50—C51—C52	7.1 (5)
C15—C16—C22—C23	76.7 (5)	C50—C51—C52—C53	150.6 (4)
C17—C16—C22—C23	−46.5 (5)	C50—C51—C52—C46	20.4 (5)
C18—C16—C22—C23	−163.8 (4)	C47—C46—C52—C53	−48.1 (5)
C15—C16—C22—C21	−157.7 (4)	C45—C46—C52—C53	75.0 (5)
C17—C16—C22—C21	79.1 (4)	C48—C46—C52—C53	−165.9 (4)
C18—C16—C22—C21	−38.3 (4)	C47—C46—C52—C51	78.3 (4)
C20—C21—C22—C23	149.7 (3)	C45—C46—C52—C51	−158.6 (4)
C20—C21—C22—C16	19.8 (4)	C48—C46—C52—C51	−39.5 (4)
C16—C22—C23—C25	176.3 (4)	C51—C52—C53—C54	178.0 (4)
C21—C22—C23—C25	55.4 (5)	C46—C52—C53—C54	−60.7 (5)
C16—C22—C23—C24	−59.0 (5)	C51—C52—C53—C55	53.9 (5)
C21—C22—C23—C24	−179.8 (3)	C46—C52—C53—C55	175.2 (4)
C22—C23—C25—C26	−175.7 (4)	C54—C53—C55—C56	59.1 (5)
C24—C23—C25—C26	58.8 (5)	C52—C53—C55—C56	−175.5 (4)
C23—C25—C26—C27	164.9 (4)	C53—C55—C56—C57	159.8 (4)
C25—C26—C27—C28	152.7 (5)	C55—C56—C57—C58	142.3 (5)
C26—C27—C28—C29	−2.5 (8)	C56—C57—C58—C59	0.3 (9)
C26—C27—C28—C30	178.1 (5)	C56—C57—C58—C60	178.1 (5)
C27—C28—C30—O2	−119.1 (5)	C57—C58—C60—O4	−119.2 (5)
C29—C28—C30—O2	61.5 (6)	C59—C58—C60—O4	58.8 (6)

### Hydrogen-bond geometry (Å, °)

**Table:**

*D*—H···*A*	*D*—H	H···*A*	*D*···*A*	*D*—H···*A*
O1—H1···O2^i^	0.84	2.11	2.807 (5)	140
O2—H2···O3^ii^	0.84	1.97	2.784 (5)	162
O3—H3···O4^iii^	0.84	2.00	2.747 (5)	148
O4—H4···O1	0.84	1.89	2.722 (4)	173

Symmetry codes: (i) −*x*+1, *y*+1/2, −*z*+1; (ii) *x*−2, *y*−1, *z*−1; (iii) −*x*+3, *y*+1/2, −*z*+2.
